# Macitentan and phosphodiesterase-5 inhibitor alone or in combination in newly diagnosed pulmonary arterial hypertension: a pooled analysis

**DOI:** 10.1016/j.jhlto.2025.100462

**Published:** 2025-12-10

**Authors:** Vallerie V. McLaughlin, Nicolas Sauvageot, Brian Hennessy, Sumeet Panjabi, Carly J. Paoli, Jörg Linder, Bjorn Bayer, Stefan Söderberg, Sean Gaine, Tobias J. Lange, Nick H. Kim

**Affiliations:** a Division of Cardiovascular Medicine, University of Michigan, Ann Arbor, MI; b Frankel Cardiovascular Center, Ann Arbor, MI; c Johnson & Johnson, Allschwil, Switzerland; d Johnson & Johnson, Titusville, NJ; e Johnson & Johnson-Cilag GmbH, Neuss, Germany; f Department of Public Health and Clinical Medicine, Cardiology and Heart Centre, Umeå University, Umeå, Sweden; g National Pulmonary Hypertension Unit, Mater Misericordiae University Hospital, Dublin, Ireland; h Department of Pulmonology, Kreisklinik Bad Reichenhall, Bad Reichenhall, Germany; i Faculty of Medicine, Department of Internal Medicine II, Regensburg University, Regensburg, Germany; j Division of Pulmonary, Critical Care, and Sleep Medicine, University of California San Diego, La Jolla, CA

**Keywords:** Pulmonary arterial hypertension, Macitentan, Phosphodiesterase-5 inhibitor, Endothelin receptor antagonist, Upfront combination therapy, Mortality, Survival

## Abstract

**INTRODUCTION:**

Upfront combination therapy with endothelin receptor antagonist (ERA) and phosphodiesterase-5 inhibitor (PDE5i) is guideline-recommended for low- or intermediate-risk pulmonary arterial hypertension (PAH). This study compared time to all-cause mortality for macitentan+PDE5i combination and each monotherapy.

**METHODS:**

Long-term patient-level data (planned follow-up ≥1 year) were pooled from four clinical trials and three observational registries. All-cause mortality data were available from adults with incident PAH initiated on macitentan 10 mg or PDE5i monotherapy, or macitentan+PDE5i. Propensity-score (PS) methods balanced key demographic and disease characteristics across cohorts. Hazard ratios (HRs) were computed from a Cox-regression model that included PS-calculated weights. Weighted Kaplan–Meier estimates with 95% confidence intervals (CI) were computed 6-monthly.

**RESULTS:**

2201 patients were included: 754 received macitentan+PDE5i (421 tadalafil, 324 sildenafil, 9 other), 654 macitentan, and 793 PDE5i (301 tadalafil, 490 sildenafil, 2 other). After weighting, characteristics were similar across cohorts. Upfront macitentan+PDE5i was associated with a 39% risk reduction of all-cause mortality versus PDE5i (HR 0.61 [95% CI 0.46–0.82]) and 32% versus macitentan (HR 0.68 [95% CI 0.37–0.95]). Reduction in all-cause mortality was 49% for macitentan+tadalafil vs tadalafil (HR 0.51 [95% CI 0.30–0.85]), 43% for macitentan+tadalafil vs macitentan (HR 0.57 [95% CI 0.37–0.88]), 31% for macitentan+sildenafil vs sildenafil (HR 0.69 [95% CI 0.45–1.0]) and 26% for macitentan+sildenafil vs macitentan (HR 0.74 [95% CI 0.46–1.17]).

**CONCLUSION:**

This large, pooled analysis suggests an observed statistical association indicating a potential survival benefit for early macitentan+PDE5i versus either monotherapy in newly diagnosed PAH.

## Graphical abstract

**Figure:**
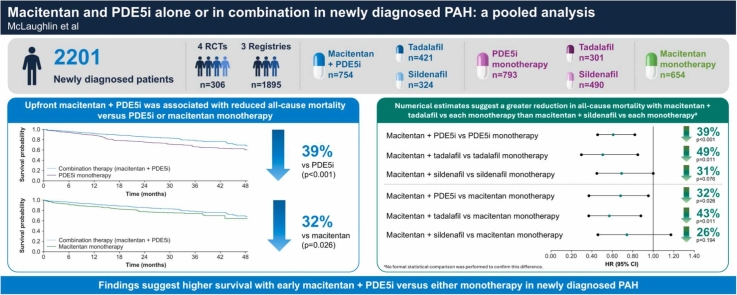

## Introduction

Upfront combination therapy with an endothelin receptor antagonist (ERA) and a phosphodiesterase-5 inhibitor (PDE5i) is widely accepted as standard-of-care treatment for pulmonary arterial hypertension (PAH).^1^ The 2022 European Society of Cardiology/European Respiratory Society guidelines recommend it for newly treated patients with PAH without cardiopulmonary comorbidities who are at low or intermediate risk.^2^ It is also recommended by an expert consensus from the 7th World Symposium on Pulmonary Hypertension^3^ based on evidence of improvements in clinical outcomes and hemodynamics from clinical studies, including AMBITION,^4^ TRITON,^5^ and OPTIMA,^6^ among others.^7^, ^8^, ^9^ Further, the recent A DUE study showed that therapy with a single-tablet combination of macitentan and tadalafil, including as upfront therapy in treatment-naïve patients, produced significantly greater improvements in pulmonary vascular resistance (PVR) compared with either monotherapy over 16 weeks.^10^, ^11^ Larger improvements in right ventricular function and structure with upfront macitentan and PDE5i combination therapy compared with macitentan monotherapy have also been demonstrated in newly diagnosed PAH in REPAIR.^12^

Real-world treatment patterns reveal a shift toward upfront combination therapy in patients newly diagnosed with PAH.^13^, ^14^, ^15^ According to the European COMPERA registry, use of early combination therapy increased from 10% in 2010 to 25% in 2019.^14^ More recently, in interim data from the ongoing EXPOSURE registry, double combination therapy was initiated in 43% of patients with incident PAH.^13^ However, many patients with PAH are still initiated on monotherapy, particularly in the United States.^16^, ^17^, ^18^, ^19^

The scientific community is still debating the risks/benefits of upfront combination therapy in low-risk PAH, where the absolute risk reduction is small.^20^ In the absence of long-term randomized controlled trials (RCTs), more evidence on hard outcomes will help to establish the role of upfront combination therapy in the standard-of-care treatment for newly diagnosed PAH in clinical practice.

A pooled analysis of patient-level data from clinical trials and registries was conducted to assess the effectiveness of early (including upfront) combination therapy with macitentan 10 mg and PDE5i in comparison with PDE5i or macitentan monotherapy in patients newly diagnosed with PAH. A primary endpoint of all-cause mortality was investigated because demonstrating the effectiveness of the dual combination of macitentan+PDE5i, relative to either monotherapy, on a long-term clinical endpoint would provide important complementary evidence to support the short-term benefits of the single-tablet combination observed in A DUE.^10^

## Methods

### Data sources and search strategy

Clinical studies and observational registries were eligible for inclusion if they contained data on all-cause mortality and hospitalization outcomes in patients with PAH following treatment with either macitentan 10 mg or PDE5i monotherapy, or their combination. They also had to meet the following criteria: (i) have access to patient-level data; (ii) have long-term data with planned follow-up ≥1 year; (iii) include only adults with PAH; and (iv) collect data prospectively after enrollment.

Four clinical trials (SERAPHIN, GRIPHON, TRITON, and REPAIR) and three observational real-world registries (REVEAL, OPUS, and EXPOSURE) met the inclusion criteria (Table 1). Full details of the studies have been published previously.^5^, ^12^, ^13^, ^21^, ^22^, ^23^, ^24^, ^25^ The OPsumit® Historical USers cohort (OrPHeUS) medical chart review was excluded because it was a retrospective study.

**Table 1:** Data Sources and Numbers of Patients Included in the Pooled Analysis, According to Treatment Cohort (Primary Analysis)

Data Sources			Pooled Analysis Population, n (%)			
Study	Design	Patients (n)	Macitentan 10 mg + PDE5i Combination Therapy	Macitentan 10 mg Monotherapy	PDE5i Monotherapy	Total
Clinical studies						
SERAPHIN (NCT00660179)^21^	Phase 3, randomized, double-blind, event-driven Macitentan (3 mg and 10 mg) vs placebo	PAH with or without stable background PAH-specific therapy (724)	23 (3)	34 (5)	21 (3)	78 (4)
GRIPHON (NCT01106014)^22^	Phase 3, randomized, double-blind, event-driven Selexipag vs placebo	PAH with or without stable ERA or PDE5i, or both (1156)	–a	–a	50 (6)	50 (2)
TRITON (NCT02558231)^5^	Phase 3b, randomized, double-blind Triple-combination (macitentan 10 mg + tadalafil + selexipag) vs double combination (macitentan 10 mg + tadalafil + placebo)	Newly diagnosed, treatment-naïve PAH (247)	118 (16)	–	–	118 (5)
REPAIR (NCT02310672)^12^	Phase 4, open-label, single-arm RV and hemodynamic outcomes Macitentan 10 mg	PAH with or without stable PDE5i (72)	42 (6)	18 (3)	–	60 (3)
Observational registry studies						
REVEAL (NCT00370214)^23^, ^24^	Multicenter, prospective, US disease registry (March 2006 to December 2009)	Newly and previously diagnosed PAH (3515)	–a	–a	238 (30)	238 (11)
OPUS^b^ (NCT02126943)^25^	Multicenter, prospective, US drug registry (April 2014 to June 2020)	PH patients newly treated with macitentan (2722)	456 (60)	413 (63)	–	869 (39)
EXPOSURE (EUPAS19085)^13^	Multicenter, prospective, European/Canadian observational study (September 2017 to ongoing)	PAH patients initiating a new PAH-specific therapy (2069^c^)	115 (15)	189 (29)	484 (61)	788 (36)
Total patients newly diagnosed with PAH			754	654	793	2201

Abbreviations: ERA, endothelin receptor antagonist; PAH, pulmonary arterial hypertension; PDE5i, phosphodiesterase-5 inhibitor; PH, pulmonary hypertension; RV, right ventricular; WHO, World Health Organization.

As the objective was to examine the specific combination of macitentan+PDE5i, patients receiving intravenous, subcutaneous, inhaled, or oral prostanoids, soluble guanylate cyclase stimulators, or an ERA other than macitentan at index date were excluded from the current study’s analysis.

a Macitentan data were not available from GRIPHON or REVEAL at the time this analysis was conducted.

b The OPUS registry was included despite having non-WHO Group 1 patients overall; however, only patients with WHO Group 1 were included for analysis per the inclusion criteria. OrPHeUS, which met some of the study’s eligibility criteria, was excluded as its data were collected retrospectively.^25^ The Phase 4 prospective OPTIMA study, which explored the effect of macitentan as upfront combination therapy with tadalafil,^6^ was not included because hospitalization had not been recorded, precluding analysis of the current study’s secondary endpoint.

c Enrollment as of 30 November 2022.

All studies were conducted in accordance with the Declaration of Helsinki and approved by international ethics committees, with written informed consent obtained from patients. As such, ethics review and approval were not required for the current analysis.

### Index date definition

The index date was aligned to be consistent with the start of the observation period. For SERAPHIN and REPAIR, this was date of study drug start; for GRIPHON and TRITON, it was randomization date; for OPUS, it was macitentan initiation date; for REVEAL, it was enrollment date; and for EXPOSURE, it was date of initiation of new PAH-specific therapy.

### Patient selection

An overview of patient selection is provided in Supplementary File S1. All randomized patients from SERAPHIN, GRIPHON, and TRITON, all patients in the REPAIR study who received treatment, and all enrolled patients in the REVEAL, OPUS, and EXPOSURE registries who were ≥18 years of age (at index date) with a recent (i.e., within 6 months of index date) diagnosis of PAH (World Health Organization [WHO] Group 1 pulmonary hypertension [PH]) were included. To ensure a comparable patient population across studies, only newly diagnosed patients were included from each trial/registry; however, this criterion did exclude a substantial number of patients from key studies, such as SERAPHIN. Patients from observational registries were required to have ≥1 follow-up visit. To specifically examine the combination of macitentan+PDE5i, patients receiving intravenous, subcutaneous, inhaled, or oral prostanoids, soluble guanylate cyclase stimulators, or an ERA other than macitentan at index date were excluded. Additionally, patients who started any PAH-specific therapy >6 months before the index date and those who died before the index date were ineligible for inclusion.

### Cohort definitions

For the primary analysis, patients were divided into three cohorts according to treatment: (i) combination therapy (macitentan+PDE5i), where both drugs were received at index date or one was received at index date and the second started within a 30-day time window from initiation of the first; (ii) macitentan monotherapy, where only macitentan was received at index date; and (iii) PDE5i monotherapy, where only PDE5i was received at index date. For all cohorts, no other PAH-specific therapies were added within a 30-day time window from index date. Patients taking their assigned medication (macitentan, PDE5i, or both) for ≤6 months before index date were eligible for inclusion, as per each trial/registry protocol.

In the United States, macitentan requires insurance pre-authorization, which can incur delays in the initiation of therapy. Therefore, a 30-day time window definition was used to capture patients who started PDE5i and macitentan treatment on the same day, as well as those undergoing rapid sequential therapy. In Europe (where most patients in EXPOSURE are enrolled^13^), macitentan does not need pre-authorization, meaning macitentan and PDE5i can be initiated together (i.e., 0-day time window). This primary analysis therefore focuses on a 30-day window for all studies except EXPOSURE, to account for these regional access differences. To assess the robustness of the primary analysis, a sensitivity analysis was also conducted, using a 30-day time window for all studies, including EXPOSURE.

### Statistical analysis

#### Approach

The primary objective of this pooled analysis was to evaluate the effect of upfront macitentan+PDE5i combination therapy compared with macitentan alone and PDE5i alone. The primary endpoint was time to all-cause mortality and the secondary endpoint was the composite of time to all-cause mortality or first all-cause hospitalization (the registries did not include adjudication, meaning PAH-related events could not be identified). Each objective was analyzed using two strategies to account for intercurrent events, defined as discontinuation of macitentan or PDE5i during the study or the start of a new PAH therapy at any time. A treatment policy strategy, which uses all data regardless of whether an intercurrent event has occurred (similar to an intent-to-treat strategy), was used for the main analysis. Using a while on treatment (WOT) strategy, data were censored as soon as an intercurrent event occurred. Subgroup analyses are outlined in Supplementary File S2. Since all analyses were exploratory and no multiplicity adjustment was made, *p*-values were generated solely for descriptive purposes and do not imply statistical significance.

#### Baseline characteristics

Key baseline characteristics (sex, age, PAH etiology, WHO functional class, 6-minute walking distance [6MWD], mean pulmonary arterial pressure, PVR, cardiac index, mean right arterial pressure, heart rate, and body mass index) were identified as clinically meaningful for the outcome. Data for N-terminal prohormone of brain natriuretic peptide were not included because measurements were not directly comparable between studies due to the use of different assays and laboratory techniques. Missing data were imputed using the Multiple Imputation by Chained Equations algorithm, resulting in the creation of multiple (57) imputed datasets (Supplementary File S3).

#### Propensity score (PS)

For each imputed dataset, differences in important baseline demographic and disease characteristics between treatment cohorts were addressed by PS weighting using the baseline characteristics described earlier; 6MWD was not included because there was a substantial proportion (34.5%) of 6MWD data missing. Sensitivity analyses showed no difference in results when 6MWD was included versus not included (data not shown). PS weighting was used because this method, unlike PS matching, allowed all patients to be retained in the analysis. Two separate PS models were developed: one comparing the combination therapy cohort with the PDE5i monotherapy cohort, and another comparing the combination therapy cohort with the macitentan monotherapy cohort. Separate models were used for the main analysis and subgroup analyses. Weights of 1 were maintained for patients in the combination therapy cohort, while weights for patients in the monotherapy cohorts were adjusted based on their likelihood of being assigned to the combination therapy. For each imputed dataset, trimming (excluding cases in the regions of non-overlap: <2% [data not shown]) and truncation (weights beyond the threshold of 10: <1% [data not shown]) were performed.

#### Estimation of combination therapy cohort effects

Weighted hazard ratios (HRs) were computed from weighted Cox-regression models for each imputed dataset and pooled. Similarly, weighted Kaplan–Meier estimates and corresponding 95% confidence intervals (CI) were computed at 6-monthly timepoints for each imputed dataset and pooled.

Statistical analyses were conducted using SAS® version 9.3 or higher.

## Results

### Study population

In total, 2201 patients with newly diagnosed PAH meeting the eligibility criteria were included in the primary analysis from four clinical studies and three observational registries. Across studies, median follow-up ranged from 13 months (REPAIR) to 41 months (REVEAL), with most studies having upper quartile follow-up between 28 and 35 months. Most data came from the OPUS (39%) and EXPOSURE (36%) registries (Table 1). Among all patients, baseline characteristics had the following number and proportion of missingness: sex (0 patients [0%]), age (0 [0%]), PAH etiology (1 [<0.1%]), WHO functional class (289 [13.1%]), 6MWD (769 [34.9%]), mean pulmonary arterial pressure (163 [7.4%]), PVR (347 [15.8%]), cardiac index (354 [16.1%]), mean right arterial pressure (325 [14.8%]), heart rate (137 [6.2%]), and body mass index (150 [6.8%]).

In the primary analysis, treatment cohorts comprised 754 patients treated with macitentan 10 mg combined with a PDE5i, 654 treated with macitentan 10 mg monotherapy, and 793 treated with PDE5i monotherapy (Table 1). The PDE5i cohort was divided into patients on tadalafil (421 combination, 301 monotherapy) and sildenafil (324 combination, 490 monotherapy). Eleven patients (9 combination, 2 monotherapy) treated with other PDE5is (e.g., vardenafil) were excluded from the PDE5i subgroup analysis. Subsequent therapy (which could include ERA, PDE5i, prostanoids or prostacyclin receptor inhibitors, and soluble guanylate cyclase inhibitors) was received by 231 (31%) patients in the combination therapy cohort (median [interquartile range] time from index date to start of therapy 238 [82−573] days), 421 (53%) patients in the PDE5i cohort (94 [15−265] days), and 244 (37%) patients in the macitentan monotherapy cohort (103 [32−207] days).

Baseline demographic and clinical characteristics for the primary analysis population were broadly similar across treatment cohorts (Table 2), with slight differences in age, sex, and PAH etiology. The combination and PDE5i cohorts had more severe WHO functional class and PVR than the macitentan cohort. In addition, diuretic use was higher in the combination cohort versus the macitentan and PDE5i cohorts, more patients in the PDE5i cohort had underlying hypertension, and underlying renal insufficiency was more prevalent in the combination and macitentan cohorts. Importantly, these baseline characteristics were similar after weighting (Table 2).

**Table 2:** Observed and Weighted Patient Characteristics at Baseline According to Treatment Cohort in the Primary Analysis

	Macitentan+PDE5i Combination vs Macitentan Monotherapy			Macitentan+PDE5i Combination vs PDE5i Monotherapy		
	Macitentan+ PDE5i Combination Therapy (n=754)	Macitentan Monotherapy Unweighted (n=645)	Macitentan Monotherapy Weighted (n=748)	Macitentan+ PDE5i Combination Therapy (n=746)	PDE5i Monotherapy Unweighted (n=786)	PDE5i Monotherapy Weighted (n=737)
Mean age (SD)	57.6 (15.06)	62.8 (13.94)	57.2 (16.21)	57.6 (15.11)	59.1 (16.82)	56.6 (16.97)
abs. SD		0.356	0.027		0.093	0.061
abs. SD(%)		57(100)	0		0	0
Male, n (%)	205 (27.2)	169 (26.2)	184 (24.7)	204 (27.4)	260 (33.0)	192 (26.0)
abs. SD		0.022	0.058		0.124	0.031
abs. SD(%)		0	0		0	0
PAH etiology, n (%)						
Idiopathic PAH	397 (52.6)	384 (59.6)	385 (51.5)	395 (53.0)	444 (56.5)	389 (52.8)
CTD	223 (29.6)	194 (30.1)	213 (28.5)	216 (29.0)	183 (23.3)	208 (28.2)
Other	134 (17.8)	66 (10.2)	149 (20.0)	134 (18.0)	159 (20.2)	140 (19.0)
abs. SD		0.207	0.077		0.136	0.029
abs. SD(%)		47(82.5)	0		0	0
WHO FC, n (%)						
I	32 (4.3)	48 (7.5)	31 (4.1)	32 (4.3)	25 (3.1)	27 (3.7)
II	175 (23.2)	223 (34.7)	173 (23.2)	174 (23.4)	209 (26.5)	192 (26.1)
III	497 (65.9)	351 (54.5)	499 (66.8)	490 (65.7)	493 (62.7)	474 (64.3)
IV	50 (6.6)	21 (3.3)	44 (5.9)	49 (6.6)	60 (7.6)	44 (5.9)
abs. SD		0.331	0.048		0.112	0.081
abs. SD(%)		57(100)	0		0	0
6MWD, m	305.8 (124.52)	306.9 (128.44)	294.3 (130.23)	306.8 (124.56)	310.5 (130.75)	298.9 (129.06)
abs. SD		0.024	0.090		0.032	0.063
abs. SD(%)		0	0		0	0
mPAP, mmHg	49.4 (12.63)	41.6 (12.37)	50.3 (14.71)	49.4 (12.65)	46.8 (13.25)	49.4 (13.54)
abs. SD		0.620	0.065		0.194	0.008
abs. SD(%)		57(100)	0		18(31.6)	0
PVR, dyn•s•cm^-5^	832.8 (397.88)	639.8 (375.90)	838.3 (436.46)	829.2 (396.80)	770.3 (469.56)	834.8 (479.03)
abs. SD		0.499	0.019		0.136	0.014
abs. SD(%)		57(100)	0		0	0
Cardiac index, L/min/m^2^	2.4 (0.82)	2.6 (0.85)	2.4 (0.79)	2.4 (0.82)	2.5 (0.90)	2.4 (0.84)
abs. SD		0.266	0.054		0.112	0.018
abs. SD(%)		57(100)	0		0	0
mRAP, mmHg	9.6 (6.34)	9.3 (5.49)	9.7 (5.96)	9.6 (6.34)	9.2 (5.98)	9.6 (5.81)
abs. SD		0.060	0.022		0.072	0.011
abs. SD(%)		0	0		0	0
Heart rate, bpm	81.3 (14.27)	78.0 (14.50)	81.8 (14.71)	81.3 (14.29)	79.8 (14.97)	80.8 (14.78)
abs. SD		0.229	0.031		0.104	0.037
abs. SD(%)		56(98.2)	0		0	0
BMI, kg/m^2^	28.7 (6.81)	30.0 (7.33)	28.8 (7.03)	28.7 (6.81)	28.0 (6.65)	28.4 (7.15)
abs. SD		0.186	0.017		0.097	0.041
abs. SD(%)		5(8.8)	0		0	0
Non-PH medication, n (%)						
Diuretics	441 (58.5)	255 (39.6)	416 (55.7)	433 (58.0)	173 (22.0)	419 (56.8)
abs. SD		0.384	0.057		0.790	0.025
abs. SD(%)		57(100)	0		57(100)	0
Ongoing comorbidities, n (%)						
Diabetes mellitus	154 (20.4)	147 (22.7)	165 (22.1)	154 (20.6)	175 (22.2)	132 (17.9)
abs. SD		0.056	0.041		0.039	0.068
abs. SD(%)		0	0		0	0
Hypertension	221 (29.3)	213 (33.1)	214 (28.6)	221 (29.6)	343 (43.7)	194 (26.3)
abs. SD		0.082	0.016		0.294	0.074
abs. SD(%)		0	0		57(100)	0
Renal insufficiency	51 (6.8)	25 (3.9)	53 (7.0)	43 (5.7)	15 (1.9)	36 (4.9)
abs. SD		0.129	0.013		0.199	0.034
abs. SD(%)		0	0		25(43.9)	0

Abbreviations: 6MWD, 6-minute walking distance; abs. SD, absolute standardized difference; BMI, body mass index; bpm, beats per minute; CTD, connective tissue disease; FC, functional class; mPAP, mean pulmonary arterial pressure; mRAP, mean right arterial pressure; PAH, pulmonary arterial hypertension; PDE5i, phosphodiesterase-5 inhibitor; PVR, pulmonary vascular resistance; SD(%), number and percentages of absolute standardized differences >0.2; WHO, World Health Organization.

For macitentan+PDE5i combination therapy and monotherapy unweighted groups: all patients were assigned a weight of 1; n is the average rounded to a full integer of the number of patients over the 57 imputed datasets. For monotherapy weighted groups: patients were re-weighted to balance the characteristics between the monotherapy cohort and the combination therapy cohort; n is the average rounded to a full integer of the sum of these weights over the 57 imputed datasets.

For unweighted and weighted data, the summary statistics were calculated as the average of the summary statistics over the 57 imputed datasets. The n values are the average, rounded to the nearest full integer, of the number of patients across the imputed datasets and therefore can be different between unweighted and weighted data.

### Time to all-cause mortality

#### Upfront macitentan+PDE5i versus PDE5i monotherapy

**Figure 1:**
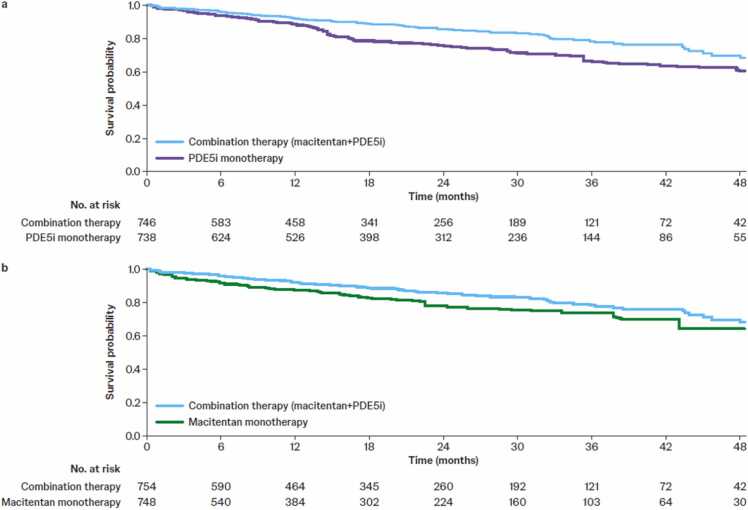
Weighted Kaplan–Meier estimates for all-cause mortality comparing (a) macitentan+PDE5i combination versus PDE5i monotherapy and (b) macitentan+PDE5i combination versus macitentan monotherapy. For macitentan+PDE5i combination therapy, all patients were assigned a weight of 1; the number at risk is the average rounded to a full integer of the number of patients who are still at risk over the 57 imputed datasets. For monotherapy groups, patients were re-weighted to balance the characteristics between the monotherapy and combination therapy cohorts; the number at risk is the average rounded to a full integer of the sum of the weights of the patients who are still at risk over the 57 imputed datasets. PDE5i, phosphodiesterase-5 inhibitor.

**Figure 2:**
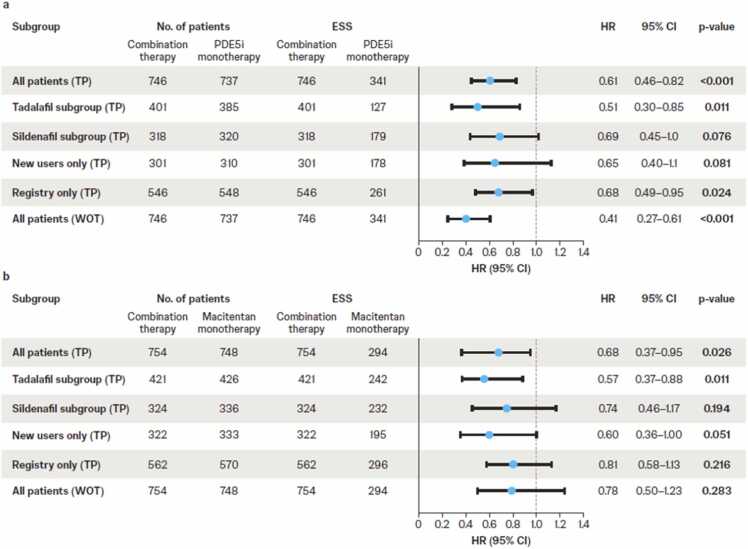
Weighted hazard ratios for time to all-cause mortality for macitentan+PDE5i combination therapy versus (a) PDE5i monotherapy and (b) macitentan monotherapy. For macitentan+PDE5i combination therapy and monotherapy unweighted groups, all patients were assigned a weight of 1; the number of patients is the average rounded to a full integer of the number of patients over the 57 imputed datasets. For monotherapy weighted groups, patients were re-weighted to balance the characteristics between the monotherapy and combination therapy cohorts; the number of patients is the average rounded to a full integer of the sum of these weights over the 57 imputed datasets. ESS represents the size of an unweighted sample with approximately the same amount of precision as the weighted sample under consideration. CI, confidence interval; ESS, effective sample size; HR, hazard ratio; PDE5i, phosphodiesterase-5 inhibitor; TP, treatment policy; WOT, while on treatment.

Upfront macitentan+PDE5i was associated with a 39% reduction in risk of all-cause mortality versus PDE5i monotherapy (HR 0.61 [95% CI 0.46–0.82], *p*<0.001; Figure 1, Figure 2). Subgroup analyses showed that macitentan+tadalafil was associated with a 49% reduction in all-cause mortality versus tadalafil monotherapy (HR 0.51 [95% CI 0.30–0.85], *p*=0.011; Figure 2a), while macitentan+sildenafil was associated with a non-significant reduction of 31% versus sildenafil monotherapy (HR 0.69 [95% CI 0.45–1.0], *p*=0.076; Figure 2a). In the WOT analysis, there was a 59% reduction in time to all-cause mortality for combination therapy versus PDE5i monotherapy (HR 0.41 [95% CI 0.27–0.61], *p*<0.001; Figure 2a). Treatment effects among new users and registry-only subgroups were of similar magnitude (Figure 2a).

#### Upfront macitentan+PDE5i versus macitentan monotherapy

Upfront macitentan+PDE5i was associated with a 32% reduction in risk of all-cause mortality versus macitentan monotherapy (HR 0.68 [95% CI 0.37–0.95], *p*=0.026; Figure 1, Figure 2). The combination of macitentan+tadalafil was associated with a 43% reduction versus tadalafil monotherapy (HR 0.57 [95% CI 0.37–0.88], *p*=0.011; Figure 2b), while the combination of macitentan+sildenafil was associated with a non-significant reduction of 26% versus sildenafil monotherapy (HR 0.74 [95% CI 0.46–1.17], *p*=0.194; Figure 2b). The subgroup analysis of new users showed similar results; however, the reduction in risk of all-cause mortality with combination therapy in the registry-only subgroup or the WOT analysis was not significant (Figure 2b).

#### Sensitivity analysis

Results from the sensitivity analysis are shown in Supplemental File S4.

### Time to all-cause mortality and hospitalization

**Figure 3:**
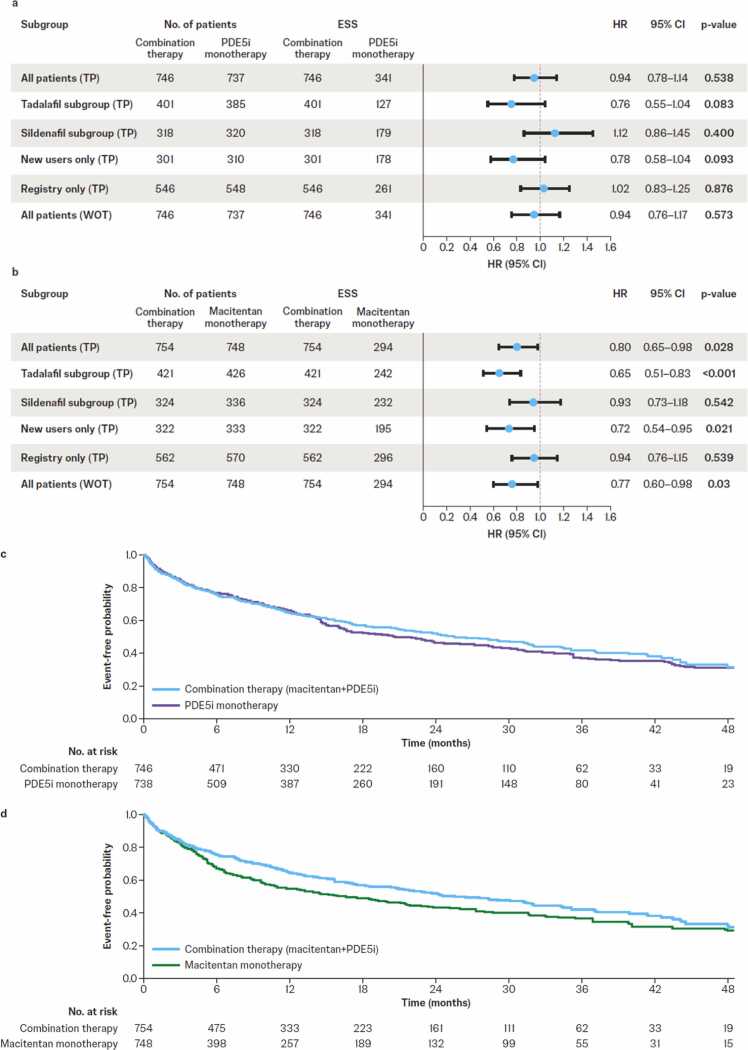
Weighted hazard ratios for time to the composite of all-cause mortality and all-cause hospitalization for macitentan+PDE5i combination therapy versus (a) PDE5i monotherapy and (b) macitentan monotherapy and weighted Kaplan–Meier estimates for all-cause mortality and all-cause hospitalization for macitentan+PDE5i combination therapy versus (c) PDE5i monotherapy and (d) macitentan monotherapy. CI, confidence interval; ESS, effective sample size; HR, hazard ratio; PDE5i, phosphodiesterase-5 inhibitor; TP, treatment policy; WOT, while on treatment.

Comparison of combination therapy with PDE5i monotherapy showed no treatment effect on the composite secondary endpoint of time to all-cause mortality or first all-cause hospitalization (HR 0.94 [95% CI 0.78–1.14], *p*=0.538; Figure 3a). However, these data must be interpreted with caution as it appears that the proportional hazards assumption was not met. Weighted Kaplan–Meier curves, which do not rely on the proportional hazards assumption, overlapped at 12 months, signifying no difference between combination therapy versus PDE5i monotherapy before 12 months and in favor of combination therapy after 12 months (Figure 3c). A moderate treatment effect was observed for combination therapy compared with macitentan monotherapy (HR 0.80 [95% CI 0.65–0.98], *p*=0.028; Figures 3b and d).

### Analyses accounting for study-level variation

Analyses to account for study-level variation were performed by adjusting for the data sources (i.e., the different trials and registries), and showed no significant differences compared with the primary results (data not shown).

## Discussion

This pooled analysis of patients newly diagnosed with PAH and treated with upfront macitentan+PDE5i combination therapy, macitentan monotherapy, or PDE5i monotherapy in clinical trials or routine clinical practice examined relative treatment effects. Patient-level data were included from three RCTs (SERAPHIN, GRIPHON, and TRITON) and one single-arm clinical study (REPAIR) alongside real-world data from three observational registries (REVEAL, OPUS, and EXPOSURE).^5^, ^12^, ^13^, ^21^, ^22^, ^23^, ^24^, ^25^ Upfront macitentan+PDE5i showed a strong treatment effect on time to all-cause mortality compared with PDE5i monotherapy (∼40–60% reduction) and a moderate treatment effect versus macitentan monotherapy (∼20–30% reduction). Reduction in all-cause mortality was 43–49% for the combination of macitentan+tadalafil versus tadalafil monotherapy and 26–31% for the combination of macitentan+sildenafil versus sildenafil monotherapy.

Several real-world studies have investigated the effectiveness of upfront combination therapy in newly diagnosed patients with PAH^14^, ^15^, ^26^; however, methodological flaws confound the interpretation of their results. A key strength of the current analysis is its scale. This was a large, pooled analysis of 2201 patients with newly diagnosed PAH initiated on macitentan+PDE5i combination therapy or macitentan or PDE5i monotherapy. Few studies have compared the effects of these different therapies in PAH. REPAIR evaluated the effect of macitentan, with or without PDE5i, on right ventricular remodeling/function and cardiopulmonary hemodynamics in 42 patients with PAH over 52 weeks.^12^ Exploratory findings indicated larger improvements in right ventricular function and structure with combination therapy versus macitentan monotherapy.^12^ Additionally, the A DUE study investigated the efficacy and safety of a single-tablet combination of macitentan 10 mg and tadalafil 40 mg compared with macitentan or tadalafil monotherapy in patients who were treatment-naïve or receiving prior monotherapy with a PDE5i or ERA.^10^ Upfront combination therapy produced significantly greater improvements in PVR after 16 weeks compared with either monotherapy.^10^ The AMBITION study also provided evidence of the benefit of combination therapy, demonstrating a lower risk of clinical failure with ambrisentan+tadalafil compared with either monotherapy in treatment-naïve patients.^4^

Meta-analyses and pooled analyses are among the highest levels of scientific evidence, particularly when synthesizing results from RCTs. While meta-analyses use statistical analyses to generate a summary estimate using effect estimates from multiple studies, pooled analyses use statistical analyses to generate summary estimates using individual patient-level data across multiple studies. In the absence of long-term event-driven RCTs comparing the effectiveness and safety of upfront combination therapy and macitentan or PDE5i monotherapy, the current pooled analysis provides the highest grade of evidence. The findings align with a subgroup analysis of the SERAPHIN study—the first RCT to demonstrate the long-term effect of macitentan versus placebo using a composite endpoint of PAH-related morbidity and all-cause mortality.^21^, ^27^ Nearly two-thirds (63.7%) of the 742 patients in SERAPHIN were taking PAH-specific background therapy, the majority of whom (97.4%) were on a PDE5i.^21^ In these patients, macitentan combined with PAH-specific background therapy reduced the risk of morbidity/mortality by 38% and risk of hospitalization by 37% versus background therapy only over a median treatment period of 115 weeks.^21^, ^27^

### Limitations

Pooled analyses have several limitations, which this study endeavored to mitigate through its design. First, combining clinical and real-world data relies on being able to account for all important measured confounding variables; however, in observational studies, frequency of visits is not defined and clinical/laboratory assessments are not mandatory, often leading to missing data. Missingness can reduce the power to detect a treatment effect, introduce bias, and result in a selected population that is not representative. In this dataset, substantial baseline data were missing for several functional, hemodynamic, and demographic parameters; therefore, missing data for baseline characteristics were imputed and PS weighting was used to ensure balance between treatment cohorts.

Secondly, despite PS weighting, the risk of confounding by indication and treatment management bias remained. Unmeasured confounding factors, such as date of diagnosis, management center, and physician’s medical preference, may have influenced differences in mortality independently of treatment effect. Furthermore, there may be substantial differences between patients enrolled in real-world registries and those enrolled in clinical trials, with those in clinical trials usually having fewer comorbidities, including cardiopulmonary comorbidities. This could limit the generalizability of our results to real-world patients, and might have contributed to the finding that patients in the registry-only subgroup had a reduction in all-cause mortality for combination therapy versus PDE5i monotherapy, but not for combination therapy versus macitentan monotherapy.

Thirdly, data were pooled from studies with variable inclusion criteria and different follow-up periods, and there may have been differences in standard-of-care management in older registries. While a WOT model was utilized to account for differences in follow-up period (the results of which complemented the treatment policy strategy), censoring of monotherapy patients when background treatment was modified potentially eliminated patients that might have benefited from the late addition of a second treatment, which could have influenced the observed treatment effect.

Fourthly, in the United States, macitentan requires pre-authorization, which can be associated with delays in combination therapy initiation. By including a 30-day time window in the sensitivity analysis, the study captured patients who initiated both drugs on the same date and those undergoing rapid sequential therapy. There were no major differences between the primary and sensitivity analyses.

Additionally, all-cause hospitalization (rather than PH-related hospitalization) was used within the secondary composite endpoint. While hospitalizations are adjudicated (often by multiple investigators) in clinical trials to determine which are PH-related, it is not possible to distinguish PH-related hospitalizations from unadjudicated hospitalizations in registry data. As the study population exhibited high comorbidity, all-cause hospitalization likely included a substantial amount of non-PH-related hospitalization, which the drugs would not be anticipated to affect. The secondary endpoint of time to all-cause mortality or first all-cause hospitalization should therefore be viewed within this context, as it might have contributed to the lack of treatment effect observed for the comparison of combination therapy versus PDE5i monotherapy, in contrast to the moderate treatment effect for combination therapy versus macitentan monotherapy. The analysis is further limited by the proportional hazards assumption not being met for the comparison of combination therapy versus PDE5i monotherapy, as indicated by overlapping Kaplan–Meier curves until 12 months.

Finally, it was not possible to categorically determine whether any patients were enrolled in both a clinical trial and a registry, and were therefore counted twice in the analysis.

## Conclusions

In line with other studies, this large, pooled analysis suggests an observed statistical association indicating a potential survival benefit of early, including upfront, macitentan 10 mg and PDE5i combination therapy on all-cause mortality compared with PDE5i or macitentan monotherapy. This analysis adds to growing evidence supporting the use of ERA+PDE5i combination therapy in newly diagnosed patients with PAH.

## Funding statement

Sponsorship for this study as well as all publication charges were funded by Johnson & Johnson. Authors employed by Johnson & Johnson were involved in the study design, analysis and interpretation of data, and writing of the manuscript.

## Data availability

Data supporting the findings of this study are available from The Clinical Trials Transformation Initiative (CTTI) database.

## Author contributions

All authors are accountable for the accuracy and integrity of the article. V.V. McLaughlin, N. Sauvageot, B. Hennessy, S. Panjabi, C.J. Paoli, J. Linder, B. Bayer, and N.H. Kim: study design, data analysis/interpretation, reviewed and critically revised the manuscript, approved the final draft. S. Söderberg, S. Gaine, and T.J. Lange: data analysis/interpretation, reviewed and critically revised the manuscript, approved the final draft.

## Declaration of Competing Interest

The authors declare the following financial interests/personal relationships which may be considered as potential competing interests: V.V. McLaughlin has received grant support from PI-Aerovate, Enzyvant/Altavant, Gossamer Bio, Keros, Sonovie, Sub-I Johnson & Johnson, and Merck/Acceleron; and scientific consulting fees from Aerovate, Apollo, CVS/Caremark, LLC, Corvista, Gossamer Bio, Johnson & Johnson, Keros, Liquidia, Merck, Morphic, Regeneron, Respira, Roivant, and United Therapeutics.

N. Sauvageot, B. Hennessy, S. Panjabi, C.J. Paoli, J. Linder, and B. Bayer are (or were at the time of analysis) employees and stockholders of Johnson & Johnson.

S. Söderberg has received compensation for consultancy or speaking for Johnson & Johnson and Merck.

S. Gaine has received honoraria and consultancy fees from Altavant, Gossamer Bio, Johnson & Johnson, and Merck.

T.J. Lange has received speaker fees and/or consultancy fees and/or financial and non-financial support for participation in scientific events and/or participated on a data safety monitoring board or advisory board for Acceleron Pharma, AstraZeneca, AOP Orphan Pharmaceuticals, Bayer, BMS, Boehringer Ingelheim, CGI Medicare, Ferrer, Gossamer Bio, Johnson & Johnson, MSD, and Pfizer.

N.H. Kim has received compensation for consultancy or speaking for Johnson & Johnson, Bayer, Merck, United Therapeutics, Gossamer Bio, and Pulnovo.
