# Supplementary material for: Macitentan and phosphodiesterase-5 inhibitor alone or in combination in newly diagnosed pulmonary arterial hypertension: a pooled analysis
Source: JHLT Open. 2025 Dec 10;11:100462. doi: 10.1016/j.jhlto.2025.100462 (PMC12818237; doi:10.1016/j.jhlto.2025.100462)
Supplement: Supplementary file 1 — Supplemental material [file mmc1.docx]

**Supplementary material**

**Supplementary File S1.** Patient Selection

|  | **SERAPHIN** | **GRIPHON** | **TRITON** | **REPAIR** | **REVEAL** | **OPUS** | **EXPOSURE** |
| --- | --- | --- | --- | --- | --- | --- | --- |
| **All patients, N** | 742 | 1156 | 247 | 87 | 3741 | 2670 | 2262 |
| **Inclusion criteria met, n (%)** | 186 (25.1) | 401 (34.7) | 245 (99.2) | 67 (77.0) | 1176 (31.4) | 1148 (43.0) | 1146 (50.7) |
| IC 1: Aged ≥18 years old at index date | 722 (97.3) | 1156 (100) | 247 (100) | 87 (100) | 3587 (95.9) | 2668 (99.9) | 2262 (100) |
| IC 2: Diagnosed ≤6 months of index date | 190 (25.6) | 401 (34.7) | 245 (99.2) | 67 (77.0) | 1218 (32.6) | 1218 (45.6) | 1241 (54.9) |
| IC 3: Group 1 PAH | 742 (100) | 1156 (100) | 247 (100) | 87 (100) | 3741 (100) | 2130 (79.8) | 2262 (100) |
| IC 4: ≥1 follow-up information |  |  |  |  | 3712 (99.2) | 2667 (99.9) | 2109 (93.2) |
| **Exclusion criteria met, n (%)** | 410 (55.3) | 182 (15.7) | 244 (98.8) | 66 (75.9) | 1025 (27.4) | 1534 (57.5) | 1272 (56.2) |
| EC 1: PAH specific therapy at index date^a^ | 701 (94.5) | 308 (26.6) | 247 (100) | 87 (100) | 1263 (33.8) | 2062 (77.2) | 1415 (62.6) |
| EC 2: Any PAH specific therapy started   6 months before index date^b^ | 419 (56.5) | 426 (36.9) | 246 (99.6) | 66 (75.9) | 1474 (39.4) | 1793 (67.2) | 1589 (70.2) |
| EC 3: Patients died before index date +30 days^c^ | 739 (99.6) | 1150 (99.5) | 245 (99.2) | 87 (100) | 3710 (99.2) | 2644 (99.0) |  |
| **Inclusion/exclusion criteria met, n (%)** | 172 (23.2) | 128 (11.1) | 242 (98.0) | 60 (69.0) | 700 (18.7) | 960 (36.0) | 973 (43.0) |
| Macitentan monotherapy cohort | 34 (4.6) |  |  | 18 (20.7) |  | 413 (15.5) | 189 (8.4) |
| PDE5i monotherapy cohort | 21 (2.8) | 50 (4.3) |  |  | 238 (6.4) |  | 484 (21.4) |
| Combination cohort | 23 (3.1) |  | 118 (47.8) | 42 (48.3) |  | 456 (17.1) | 115 (5.1) |
| Not attributed to a cohort | 94 (12.7) | 78 (6.7) | 124 (50.2) |  | 462 (12.3) | 91 (3.4) | 185 (8.2) |
| **Patients included in the analysis, n (%)** | 78 (10.5) | 50 (4.3) | 118 (47.8) | 60 (69.0) | 238 (6.4) | 869 (32.5) | 788 (34.8) |

Abbreviations: EC, exclusion criterion; ERA, endothelin receptor antagonist; IC, inclusion criterion; PAH, pulmonary arterial hypertension; PDE5i, phosphodiesterase-5 inhibitor.

^a^Specific PAH therapy includes intravenous, subcutaneous, inhaled, and oral prostanoids, soluble guanylate cyclase stimulators, and ERAs other than macitentan.
^b^Specific PAH therapy includes those listed in EC 1 and macitentan+PDE5i.
^c^Does not apply to EXPOSURE.

**Supplementary File S2.** Subgroup Analyses

Subgroup analyses using the treatment policy strategy were performed according to PDE5i received (tadalafil or sildenafil), in new users and in patients from observational registries (“registry only”). For patients receiving combination therapy, new users were defined as those who started both macitentan and PDE5i at index date or one of the two was received at index date with the second added within 30 days of the first. For those receiving monotherapy, new users were defined as those who started monotherapy at index date.

**Supplementary File S3.** Missing Data Imputation

Missing data were imputed using the Multiple Imputation by Chained Equations algorithm. The number of imputed datasets was computed at 57 as the next full integer value of FIC x 100 where FIC (fraction of incomplete cases) is the fraction of patients who had at least one missing value for the set of key baseline characteristics defined in the statistical analysis section of the article. Convergence of the algorithm was assessed by checking that a stationary process has been reached, which can be visually observed if the mean and the standard deviation (or proportions for categorical variables) remain relatively constant, and that there appears to be an absence of any sort of trend. Trace plots showed that the imputation models converged.

**Supplementary File S4.** Sensitivity Analysis

Timing for adding the second agent in the combination regimen may vary by region. Thus, a sensitivity analysis was conducted using a 30-day time window for EXPOSURE. In this analysis, sample size for the combination group increased by 112 patients to 866 with the loss of 62 patients from the macitentan monotherapy group and 156 from the PDE5i monotherapy group. The hazard ratio for time to all-cause mortality was 0.64 (95% confidence interval [CI] 0.48–0.86; *p*=0.003) for combination therapy versus PDE5i monotherapy and 0.84 (95% CI 0.59–1.18; *p*=0.312) for combination therapy versus macitentan monotherapy.

**Supplementary File S5.** Plain Language Summary

Early (including upfront) combination therapy with an endothelin receptor antagonist (ERA) and a phosphodiesterase-5 inhibitor (PDE5i) is guideline-recommended as a standard-of-care treatment for patients with lower- or intermediate-risk pulmonary arterial hypertension (PAH). These recommendations were made based on two clinical trials (AMBITION and TRITON); however, these studies were unable to determine whether upfront combination therapy had an effect on outcomes of survival for patients. To address this information gap, time to all-cause mortality was compared between upfront macitentan and PDE5i combination therapy versus either monotherapy. The analysis pooled/combined long-term, patient-level information from four trials (SERAPHIN, GRIPHON, TRITON and REPAIR) and three observational registries (REVEAL, OPUS and EXPOSURE) where data relevant for assessing all-cause mortality were available from adult patients with PAH who initiated with either macitentan 10 mg or PDE5i monotherapy, or their combination (macitentan+PDE5i). Patients were grouped according to their first treatment type after diagnosis of PAH: a combination of macitentan+PDE5i (754 patients), macitentan monotherapy (654 patients), or PDE5i monotherapy (793 patients). Statistical weighting methods were used to balance key patient characteristics in the monotherapy groups to match those of the combination group so that a fair comparison could be made. In total, 2201 patients were identified for inclusion in the study analysis. After the weighting, characteristics were broadly similar between the cohorts. Upfront treatment with combination macitentan+PDE5i was associated with a 39% risk reduction of all-cause mortality versus PDE5i monotherapy and a 32% risk reduction versus macitentan monotherapy. Overall, the results suggest an association that may indicate a potential survival benefit of early (including upfront) macitentan+PDE5i combination therapy compared with either monotherapy in patients newly diagnosed with PAH.
